# Functionalized Nanoplastics (NPs) Increase the Toxicity of Metals in Fish Cell Lines

**DOI:** 10.3390/ijms22137141

**Published:** 2021-07-01

**Authors:** Carmen González-Fernández, Francisco Guillermo Díaz Baños, María Ángeles Esteban, Alberto Cuesta

**Affiliations:** 1 Immunobiology for Aquaculture Group, Department of Cell Biology and Histology, Faculty of Biology, Regional Campus of International Excellence “Campus Mare Nostrum”, University of Murcia, 30100 Murcia, Spain; carmen.gonzalez1@um.es (C.G.-F.); aesteban@um.es (M.Á.E.); 2 Department of Physical Chemistry, Faculty of Chemistry, Regional Campus of International Excellence “Campus Mare Nostrum”, University of Murcia, 30100 Murcia, Spain; fgb@um.es

**Keywords:** gilthead seabream, cell lines, nanoplastics, metals, fish

## Abstract

Nanoplastics (NPs) are one of the most abundant environment-threatening nanomaterials on the market. The objective of this study was to determine in vitro if functionalized NPs are cytotoxic by themselves or increase the toxicity of metals. For that, we used 50 nm polystyrene nanoparticles with distinct surface functionalization (pristine, PS-Plain; carboxylic, PS-COOH; and amino PS-NH_2_) alone or combined with the metals arsenic (As) and methylmercury (MeHg), which possess an environmental risk to marine life. As test model, we chose a brain-derived cell line (SaB-1) from gilthead seabream (*Sparus aurata*), one of the most commercial fish species in the Mediterranean. First, only the PS-NH_2_ NPs were toxic to SaB-1 cells. NPs seem to be internalized into the cells but they showed little alteration in the transcription of genes related to oxidative stress (*nrf2*, *cat*, *gr*, *gsta*), cellular protection against metals (*mta*) or apoptosis (*bcl2*, *bax*). However, NPs, mainly PS-COOH and PS-NH_2_, significantly increased the toxicity of both metals. Since the coexistence of NPs and other pollutants in the aquatic environment is inevitable, our results reveal that the combined effect of NPs with the rest of pollutants deserves more attention.

## 1. Introduction

Because of their exceptional properties, including high durability, plastics are extensively produced, and parts of them are continuously discharged into the oceans [1]. Over the last decade, nanoplastics (NPs), particles smaller than 100 nm, have also been identified in aquatic environments [2]. They are known to have a high surface area/volume ratio, which makes them highly reactive [3]. Likewise, the risk of NPs is linked to their intrinsic features: surface charge, size, shape, functionalization, and coating [4] as well as to the physicochemical parameters of the surrounding media (pH, temperature and ionic concentration) and the presence or absence of natural colloids [5]. In a natural environment, NPs are polydisperse and present an asymmetrical shape and an inhomogeneously charged surface [6]. Mainly due to oxidation, NPs present negatively charged carboxylate surfaces [2,7].

Several studies have revealed the impact of NPs on a wide range of aquatic organisms like marine bivalves and zebrafish (*Danio rerio*), which are commonly used as model organisms [8,9]. In general, reports indicate that NPs harm reproduction, fertilization, embryogenesis, neuronal and locomotor activities and immunity, provoking oxidative stress [9,10,11,12]. In addition, plastic particles can act as vectors for pollutants including polycyclic aromatic hydrocarbons (*PAHs*), polychlorinated biphenyls (*PCBs*), pesticides, pharmaceuticals and heavy metals [13,14,15,16]. This fact opens a new concern about microplastic (MP) and NP toxicity. Under laboratory conditions, some studies have shown that MPs can increase bioaccumulation and toxicity of some pollutants such as mercury (Hg), producing oxidative stress in European sea bass (*Dicentrarchus labrax*) [17], or benzo[a]pyrene (BaP), which produces lethal effect in the embryos of Japanese medaka (*Oryzias latipes*) [18]. However, other studies showed that MPs decreased the toxicity of *PAHs* in zebrafish [19]. There is still scarce understanding of the rules governing the combination of NP toxicity with pollutants and their mechanisms. Even if they have the same chemical composition, MPs and NPs behave differently in natural environments, with size being one of the key factors for aggregation, pollutant adsorption and toxicity [20], as the recent capacity of NPs to adsorb metals has demonstrated [21,22]. The bioaccumulation of metals in aquatic organisms, particularly fish, implies a hazard for human health because they are one of the main sources of toxic trace elements [23].

Bearing all this in mind, the main objective of this work was to use a brain-derived fish cell line (SaB-1) as a model to investigate the potential impact of NPs, and whether they can alter the toxicity of metals, [24]. The use of fish cell lines is becoming more important as a research resource, both to gain knowledge and to obtain tools that can be used in aquaculture [25,26]. Specifically, we tested three nanoplastics made of polystyrene (PS) with different surface coatings: without functionalization (PS-Plain) or coated with carboxylic groups (PS-COOH) and amino groups (PS-NH_2_). Since we worked with a brain fish cell line, we decided to focus on two metals: arsenic (As, which is considered a metalloid) and methylmercury (MeHg), both of which are extremely toxic to marine organisms and highly bioaccumulated [27]. Methylmercury is a potent neurotoxicant and accumulates in fish brains [28]. Similarly, fish exposed to As have shown immunotoxicity and apoptosis [29]. *In vitro*, these metals caused cytotoxicity (LC_50_ for MeHg and As of 0.02 and 0.018 mM, respectively), oxidative stress and cell death in fish brain cell lines [30]. This study attempts to help understand the ecological risks of NPs.

## 2. Results and Discussion

### 2.1. Cell Culture Medium Strongly Impacts NP Characteristics

**Figure 1:**
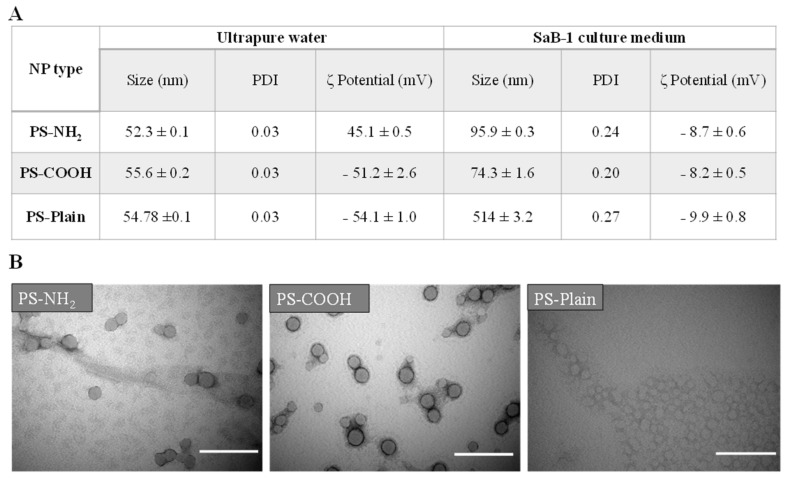
Characterization of the functionalized polystyrene (PS) nanoplastics (NPs). (**A**) Size (nm), polydispersity index (PDI) and zeta (ζ) potential mean values of the functionalized NPs, measured by DLS, ± SEM (*n* = 3) are presented. (**B**) Representative transmission electron microscopy images of the functionalized polystyrene NPs in a complete culture medium. Scale bar: 200 nm.

Fish cell lines are excellent models for studying toxicity mechanisms [25] even though the culture medium strongly influences nanoparticle behavior [31,32,33]. Using particles from a previous study [34], our data from the dynamic light scattering (DLS) analysis showed an expected size of NPs in ultrapure water for PS-NH_2_, PS-COOH and PS-Plain with sizes of 52.3 ± 0.1, 55.6 ± 0.2 and 54.8 ± 0.1 nm, respectively (polydispersity index (PDI) < 0.2), further confirmed by TEM analysis (Figure 1). In the cell culture medium, both functionalized NPs increased in size but maintained the nanoscale (<100 nm) showing sizes of 95.9 ± 0.3 nm for PS-NH_2_ and 74.3 ± 1.6 nm for PS-COOH, whereas PS-Plain NPs increased to the microscale with 514 ± 3.2 nm (PDI > 0.2) (Figure 1). By contrast, when resuspended in seawater only PS-NH_2_ remained nanosized (67.9 ± 0.8 nm) while PS-COOH and PS-Plain increased significantly reaching microsizes around 4 µm [34]. Regarding the ζ potential, all particles displayed the expected charge in UW, being PS-NH_2_ positively charged (45.1 ± 0.5 mV) and PS-COOH and PS-Plain negatively charged (−51.2 ± 2.6 and −54.1 ± 1.1, respectively). However, in the culture medium, all the particles reduced their ζ-potential and showed a similar negative charge (Figure 1), very close to the medium alone (−9.1 ± 1.0 mV) due to its ionic strength and the lack of accurate measurement of the ζ potential [35] according to the ZETASIZER manual.

We observed that NPs were more stable in a cell culture medium than in seawater, displaying nano-sizes except for PS-Plain. In natural environments, not only the presence of divalent cations but also the presence of organic matter can affect NP size promoting aggregation [36,37,38]. Molecules from water can form an environmental coating, or “eco-corona” around NPs as previously described [5,32]. Nevertheless, a similar process can occur at the cellular level. When NPs are placed into a biological environment, their surface becomes coated with a variety of biomolecules forming a “biocorona” [39]. The SaB-1 culture medium was supplemented with 10% FBS, and there seemed to be a direct and proportional correlation between the quantity of FBS in the medium and NP aggregation [40]. In fact, it seemed that FBS had a much greater effect on NPs size than the rest of the components of the culture medium and that this “bio-corona” greatly affects toxicity and behavior distribution in biological matrices [39]. As previously reported, the increase in NP size in our study was probably linked to the adsorption of proteins and lipids in the medium [39]. The slight aggregation observed in PS-NH_2_ and PS-COOH are in agreement with previous studies carried out on fish cells lines and PS-NPs [40], which could have had an impact on the toxicity of particles because they are small enough to cross biological membranes.

### 2.2. Surface Functionalization Determines Toxicity of Nanoplastics in the SaB-1 Cell Line

**Figure 2:**
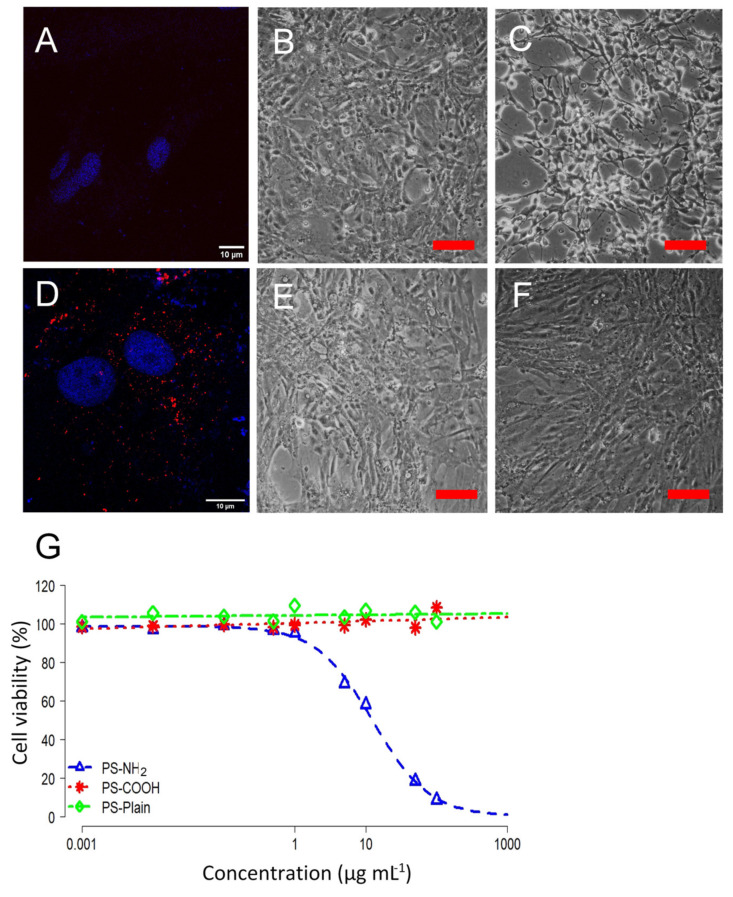
Cytotoxicity of SaB-1 cells exposed to functionalized polystyrene nanoplastics (PS-NH_2_, PS-COOH and PS-Plain) for 24 h. Representative confocal fluorescence microscopy images of unexposed SaB-1 cells (**A**) or exposed to 12 μg mL^−1^ of red PS-COOH for 24 h (**D**). Blue and red fluorescence indicate cellular DNA (DAPI) and PS-COOH (Micromer^®^-redF), respectively. Scale bar is fixed at 10 µm. Representative phase contrast microscope images of SaB-1 cell cultures unexposed (**B**; Control) or exposed for 24 h to 12 µg mL^−1^ of PS-NH_2_ (**C**), PS-COOH (**E**) or PS-Plain (**F**). Scale bar represents 100 μm. (**G**). Cytotoxicity curves of SaB-1 cells exposed to functionalized polystyrene nanoplastics. Percent cell viability is calculated as percentage of the control or unexposed cells (*n* = 3) and lines represent the fitted curves.

On the other hand, it has been widely suggested that MPs show no or low toxicity because they are not able to cross cellular membranes [41]. PS-NPs are able to permeate into lipid membranes [42] because particle size one of the key factors determining the uptake pathway [43]. In fact, the optimum size for NPs internalization and uptake rate is 50 nm [43]. Once NPs cross the membrane core, they might alter membrane structure, reduce molecular diffusion and soften the membrane, which can severely affect cellular functions [44]. In this study, we determined if the PS-NPs could be internalized in SaB-1 cells. Confocal microscopy images of SaB-1 cells exposed to red fluorescent PS-COOH as model particles clearly demonstrates that these NPs are internalized and mainly localize in the cytoplasm (Figure 2A,D), as previously reported [45]. NP internalization is suggested to be by clathrin-mediated endocytosis, macropinocytosis or phagocytosis, in which the plastic particle size, surface functionalization or even the cell type has an important cell-penetrating role [46]. Further and deeper studies should be carried out to ascertain the internalization strategies and factors limiting cellular membrane interactions with plastic particles in fish models.

Despite the fact that a strong research effort was done during the last decade, real environmental concentrations of NPs are still unknown. It is estimated that, for particles around 50 nm, the predicted environmental concentration could be below 0.015 µg mL^−1^ [47]. In this work, to identify the potential effects of NPs on cell viability, SaB-1 cells were exposed for 24 h to NP concentrations ranging from 0.001 µg mL^−1^, close to predictive environmental scenarios, to 100 µg mL^−1^. Compared to unexposed cells (Figure 2B), SaB-1 cells exposed to PS-NH_2_ displayed signs of toxicity including the formation of vacuoles and a reduction in cell viability (Figure 2C), while this was not observed in cultures exposed to PS-COOH or PS-Plain (Figure 2E, 2F). Accordingly, the MTT viability test also demonstrated that only PS-NH2 resulted toxic for SaB-1 cells and the LC_50_ was established at 12 µg mL^−1^ (Table 1, Figure 2G), which is consistent with the microscopy observations. When cytotoxicity data of SaB-1 cells exposed to the different functionalized NPs were analysed by one-way analysis of variance (ANOVA) they showed a significant effect (*p* = 0.0061). Then, Tukey’s comparison of means between NPs showed that cells exposed to PS-NH_2_ showed a statistically significant lower cell viability when compared to cells exposed to PS-COOH (*p* = 0.021) or PS-Plain (*p* = 0.0096), while no significant differences between cells exposed to PS-COOH and PS-Plain (*p* = 0.9035) were observed.

In general, the toxicity of NPs is dependent on its functionalization and cell type [41]. However, higher toxicity w been observed upon exposure to PS-NH_2_ nanoplastics than other type of functionalized NPs in different cell lines due to the strong electrostatic interaction between cationic NPs and the phosphate groups of the cell membrane [43]. In mammalian cells, both PS-NH_2_ and PS-COOH reduced cell viability compared to PS-Plain, mainly due to ionic interactions among the functionalized PS-NPs and the cell membrane [41]. These results highlighted that the toxicity of NPs greatly depends on the species and the tissue originating the cell line, as previously reported [48]. In fact, it seems that fish brain cell lines are more sensitive to NP exposure than other fish cell lines [18,39,47]. Regarding the SaB-1 cell line, PS-NH_2_ affected not only cell viability but also cell morphology, which supports the fact that positively charged NPs disrupt the integrity of cellular membranes and lead to an increase in toxicity.

### 2.3. Exposure to PS-NH_2_ Produce Oxidative Stress and Apoptosis at Transcriptional Level

**Figure 3:**
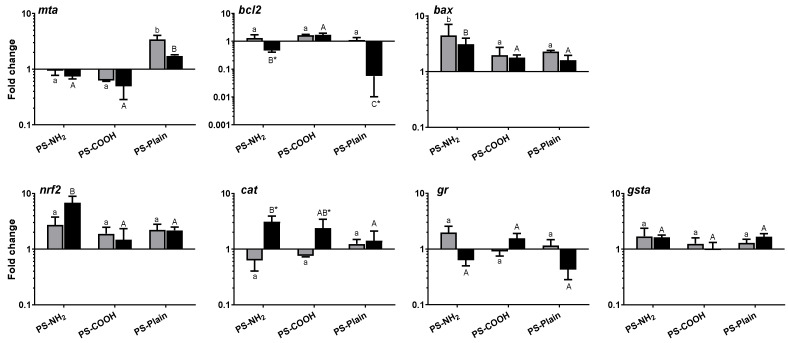
Transcriptional profile of SaB-1 cells exposed to 1 (grey bars) or 12 (black bars) µg mL^−1^ of functionalized polystyrene nanoplastics (PS-NH2, PS-COOH and PS-Plain) for 24 h. Data are presented as mean fold change of the relative gene expression in NP-exposed cells compared to unexposed cells ± SEM (*n* = 3). Different lowercase and uppercase letters denote significant differences between functionalized NPs at 1 µg mL^−1^ or 12 µg mL^−1^, respectively whilst significant differences between the two concentrations are noted by asterisks, according to ANOVA and Tukey’s post-hoc tests (*p* < 0.05). PS, polystyrene; NP, polystyrene nanoplastics; *mta*, Metallothionein A; *bcl2*, Bcl-2-associated X protein; *bax*, B-cell lymphoma 2; *nrf2*, Nuclear factor (erythroid-derived 2)-like 2; *cat*, Catalase; *gr*, Glutathione reductase; *gsta*, Glutathione S-transferase A.

Although the concentrations of PS-NPs used in this work are higher than those predicted in the environment, they allowed us to assess potential toxicological responses to, and the mechanisms of, NPs in highly polluted scenarios. Thus, to better understand these toxicity mechanisms and their functionalization, we analysed the expression of several genes related to metal protection (*mta*), apoptosis (*bcl2*, *bax*) and oxidative stress (*nrf2*, *cat*, *gr*, *gsta*) in SaB-1 cells exposed to either 1 (sublethal) or 12 µg mL^−1^ (the LC_50_ for PS-NH_2_) dosages of functionalized NPs (Figure 3). Overall, exposure to NPs produced some transcriptional changes and most of the responses depended on the NP surface functionalization more than on the dosage. In fact, the two-way-ANOVA revealed that the dosage of NPs produced significant effects in the transcription of *mta*, *bcl2*, and *cat* genes while the functionalization did in *mta*, *bcl2* and *nrf2* (Table 2). Thus, PS-NH_2_ significantly increased the expression of *nrf2*, *cat* and *bax* genes compared to the controls (Figure 3). These data suggest that SaB-1 cells exposed to PS-NH_2_ suffered oxidative stress. In this sense, reactive oxygen species (ROS) production is one of the more recognized effects of NPs on isolated cells [9,10,36,48] and has been registered in both mammalian [49,50,51,52] and fish cell lines [18,39,47]. Our data also showed that exposure to PS-NH_2_ increases the transcription of pro-apoptotic (*bax*) genes and decreases that of anti-apoptotic (*bcl2*), what creates a balance favoring apoptosis. PS-NH_2_ was the only NP tested that produced cytotoxicity, probably by apoptosis, likely due to oxidative imbalance. This relation of oxidative stress and apoptosis in cell lines exposed to NPs has been demonstrated in mammals [45], but it deserves further characterization for aquatic organisms. It was also surprising, due to its high toxicity, that PS-NH_2_ did not produce stronger effects at the genetic level in SaB-1 cells. The toxicity of PS-NH_2_ has been associated with the interaction of positive amino-groups with the negatively charged lipid membranes [42,43]. However, the low ζ-potential registered for these NPs may indicate the formation of biocorona–nanoparticle complexes. The presence of serum may protect cells from the damage induced by the cationic charges of bare nanoparticles as has been demonstrated [53].

By its side, exposure to PS-COOH particles failed to produce any significant alteration in the gene expression of SaB-1 cells whilst PS-Plain significantly up-regulated the transcription of *mta* and down-regulated *bcl2* gene expression compared to the controls (Figure 3). This was not surprising since *mta*, considered the biomarker for detoxification of toxic metals, has also other cellular functions including modulation of intracellular redox balance, anti-inflammatory processes, free-radical scavenging, and protection of neurons against neuronal lesions [54]. Perhaps, this *mta* protected neuronal SaB-1 cells by preventing certain low and transitory apoptosis, suggested by the decreased anti-apoptotic *bcl2*, since no cytotoxicity was detected after 24 h of exposure. We recall that even both PS-COOH and PS-Plain present negative charges, NPs showed different aggregation, which could impact cells differently. Interestingly, very little changes were observed when the NP concentrations were compared. Only a decrease in *bcl2* and an increase in *cat* gene expression with the highest NP concentration was detected.

### 2.4. The Presence of Nanoplastics Increases the Toxicity of Metals

**Figure 4:**
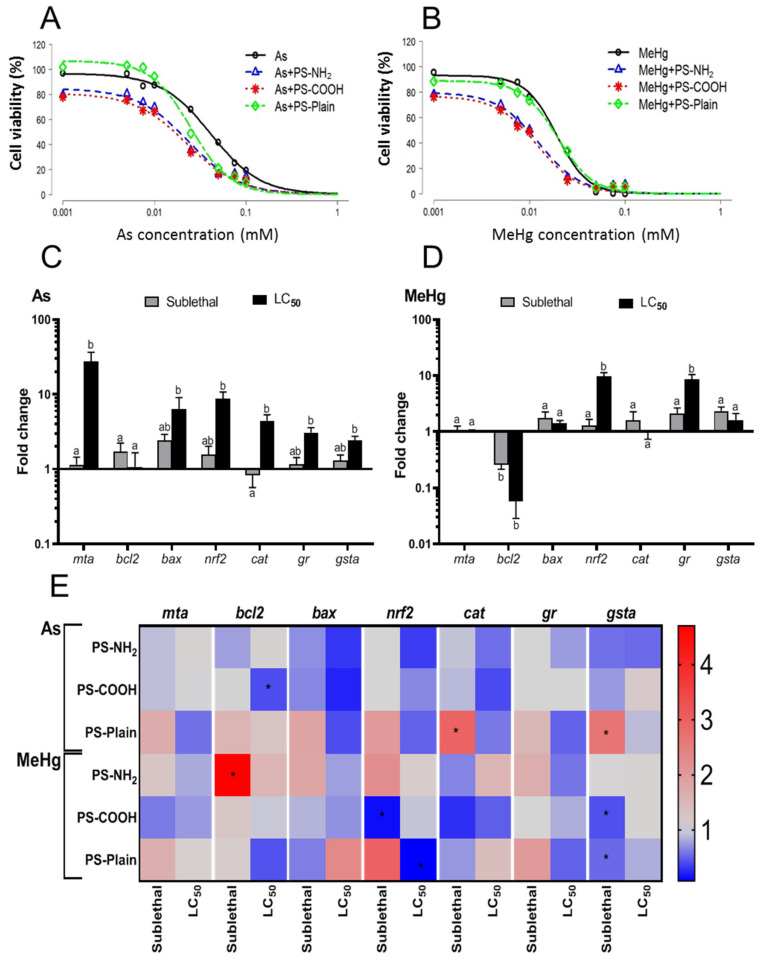
Combined effects and interactions between functionalized NPs and metals As and MeHg. Cytotoxicity curves of SaB-1 cells exposed to metals As (**A**) and MeHg (**B**) alone or in combination with 1 µg mL^−1^ of functionalized polystyrene nanoplastics (PS-NH_2_, PS-COOH and PS-Plain) for 24 h. Percent of cell viability (*n* = 3) is calculated as a percentage of the control or unexposed cells while lines are the fitted curves. The transcriptional profile of SaB-1 cells exposed to sublethal (grey bars) or LC_50_ (black bars) dosages of metals As (**C**) and MeHg (**D**) alone for 24 h. Data are presented as mean fold change of the relative gene expression in metal-exposed cells compared to unexposed cells ± SEM (*n* = 3). Differences between concentrations are marked with different letters according to ANOVA and Tukey’s post-hoc tests (*p* < 0.05). Heat-map of the transcriptional profile of SaB-1 cells exposed to sublethal or LC_50_ dosages of metals (As or MeHg) in combination with 1 µg mL^−1^ of functionalized polystyrene nanoplastics (PS-NH_2_, PS-COOH and PS-Plain) (**E**) for 24 h. Mean fold change of the transcription in SaB-1 cells exposed to metals combined with NPs respect to the transcription in SaB-1 cells exposed to metals alone is presented (*n* = 3). Asterisks denote statistically significant differences between cells exposed to metals alone and cells exposed to combined metals and NPs. PS, polystyrene; NP, polystyrene nanoplastics; *mta*, Metallothionein A; *bcl2*, Bcl-2-associated X protein; ***bax***, B-cell lymphoma 2; *nrf2*, Nuclear factor (erythroid-derived 2)-like 2; *cat*, Catalase; *gr*, Glutathione reductase; *gsta*, Glutathione S-transferase A.

The toxicity of As and MeHg to fish cell lines or specimens has already been demonstrated. Our data show that SaB-1 cells were also susceptible to both metals in a dose-dependent manner, for which reliable LC_50_ values could be calculated (Figure 4A,B, Table 1). MeHg was significantly more toxic than As (Table 1) at about half of its LC_50_ values. Previous studies showed comparable levels of toxicity for As and MeHg to seabream or brain cell lines [30,55] with similar LC_50_ values as the ones reported in this study. In addition, two-way ANOVA showed a marked effect for the metals in the transcription of *mta*, *bcl2*, *bax* or *gr* while the dosage affected *mta*, *bcl2*, *nrf2* and *gr* (Table 2). However, when we analysed the metals separately, we found a significant up-regulation of *mta*, *bax*, *nrf2*, *cat*, *gr* and *gsta* gene expression in SaB-1 cells exposed to the LC_50_ of As compared to controls (Figure 4C). In contrast, MeHg exposure produced fewer and lower effects than As: increasing the transcription of *nrf2*, *gr* and *gsta* but decreasing that of *bcl2* (Figure 4D). These data suggested that both As and MeHg induced oxidative stress and apoptosis in SaB-1, which was in agreement with previous studies on other fish cell lines [30,55].

Particular features of NPs could facilitate the adsorption of metals [21,22], so we aimed to determine if the presence of NPs, and their functionalization could alter the toxicity of As and MeHg. In general, co-exposure to NPs increased cytotoxicity being the toxicity of As significantly decreased from LC_50_ of 0.041 when exposed alone, to LC_50_ values of 0.023–0.025 when exposed in combination with functionalized NPs (Figure 4A, Table 1). In the case of MeHg, the LC_50_ was unaltered by the combination with PS-Plain but significantly decreased from 0.019 to 0.012 and 0.013 mM when combined with PS-COOH and PS-NH_2_ (Figure 4B, Table 1), respectively. There are several mechanisms by which NPs can bind to metals: i) direct adsorption of cation complexes onto charged sites or neutral regions of the surface of the particulate plastic, ii) adsorption onto hydrous oxides, or iii) co-precipitation [21]. In this sense, particle size played an important role in metal adsorption: larger particles presented lower values of bound metals [56], which made NPs particularly good contaminant vectors because of their high surface–volume ratio [57]. This was consistent with our results since PS-Plain, which showed the highest aggregation, reached sizes >500 nm and had no effect on MeHg toxicity compared to functionalized NPs that stayed nanosized. We hypothesized that the increased metal toxicity observed in presence of functionalized NPs was due to the higher adsorption of metals into the NPs thanks to their small size and high surface area–volume ratio. Since similar toxicities were registered for metals combined with PS-NH_2_ or PS-COOH, with no statistical differences among plastics, we assumed that metal–NP interaction was more affected by the plastic’s size than its charge. Although characterizing the physical–chemical interaction of plastic and metal was not the objective of this work, it should be interesting to evaluate the interactions of NP–metals for metal bioavailability, effective concentrations or adsorption/desorption kinetics. In consequence, the authors considered that further studies are needed to assess the interaction of metals and nanoplastics in the ocean to reveal their association under natural conditions and their impact on living organisms. As far as we know, in a natural environment, the adsorption of metals to MPs has been revealed to be relatively rapid thanks to their hydrophobic surface. Metals preferentially bind to beached MPs with respect to virgin MPs probably due to the presence of biofilms and irregular surfaces. However, they also bind virgin MPs with higher sorption behavior in charged or polar plastics [58]. Similar processes and effects might be expected for environmental NPs. Thus, the formation of the biocorona not only influenced NP characteristics but may also affect metal adsorption kinetics and capacity.

To unravel the potential mechanisms responsible for the increased toxicity of metals by NPs we also evaluated gene expression. Overall, the three-way ANOVA revealed that functionalization of NPs has a significant impact in the transcription of *nrf2* whilst the metal affected *mta*, *bcl2* and *gr* (Table 2). As expected, the dosage made the greatest difference. Interestingly, the interaction between metal and NP was close to significance for the transcription of *bcl2* (*p* = 0.065), *nrf2* (*p* = 0.084) and *gsta* (*p* = 0.078), but only the three factors (NP, metal and dosage) produced a significant interaction in the transcription of *nrf2*, the master transcription factor of antioxidant enzymes. We then evaluated the influence of NP co-exposure to metal-induced gene expression in the SaB-1 cells (Figure 4E). The combination of As with PS-COOH or PS-NH_2_ produced no effect or a reduction in the gene expression when compared to As alone. In sharp contrast, As combination with PS-Plain resulted in increased expression in all genes compared to the metal alone, mainly when the sublethal dose of As was combined (Figure 4E). 

Regarding MeHg, combination with PS-NH_2_ led to a significantly increased *bcl2* transcription when compared to metal alone, whilst the effect was significantly decreased for *nrf2* and *gsta* in combined exposure with PS-COOH or PS-Plain (Figure 4E). These transcriptional data somehow contradicted the cytotoxicity observed in the MTT test. In fact, gene data obtained from the co-exposure suggested that SaB-1 cells exposed to metal and NP showed lower oxidative stress and apoptosis than those exposed to the metal alone; however, the cells died in greater numbers. This may be related with the ability of NPs to bind metals making them less available to the cells. However, it is also possible that the gene expression decreased because the percentage of living cells dropped from 50% with metal alone to around 20% when combined with the NPs, resulting in less representation of genes. 

In this respect, strikingly, when the sublethal dosages of metals were combined with NPs we found some increments in genes related to oxidative stress and apoptosis that could explain the decrease in cell viability. This implied that when the combined effects were studied not only the endpoints but also their kinetics are worthy of evaluation. The literature shows unequal effects on metal toxicity when combined with MPs/NPs. For example, in sea bass juveniles, co-exposure of mercury in combination with MPs produced higher toxicity including increased mercury (Hg) bioaccumulation, neurotoxicity, oxidative stress and changes in the activities of energy-related enzymes [17,59]. An additive effect was also observed in co-exposure to chromium(VI) (Cr) in early juveniles of the common goby (*Pomatoschistus microps*), which decreased their predatory performance and inhibited AChE activity [60]. However, other studies revealed lower toxicity or no effect of metal and MP co-exposure. Thus, MPs did not influence the uptake and toxicity of silver (Ag) in zebrafish [61] while an antagonistic interaction with cadmium (Cd) was observed in the common goby juveniles [62]. In this regard, the ability of contaminants to be adsorbed to plastics may depend on the plastic’s features and the type of contaminant [63], which directly influences its combined toxicity. Other studies carried out with cell lines have revealed that NPs seem to reduce the cytotoxicity of pharmaceuticals [40,48], whereas they failed to alter the toxicity of BaP and 3-nitrobenzanthrone (*3-NBA*) [64]. In our study, the presence of functionalized NPs increased the toxicity of metals measured using the MTT technique though little alteration at the transcriptomic level was achieved. These findings were valuable for developing predictive toxicity systems and uncovering the potential adverse impact of PS–NPs on fish either alone or combined with other pollutants. Because seabream is an important species for human consumption, establishing the SaB-1 seabream brain cell line as a toxicological model might provide important information on the potential risks of pollutants not only at the ecotoxicological level but also from an economic and human health point of view.

## 3. Materials and Methods

### 3.1. Cell Culture

SaB-1 is a neuronal-like stem cell line derived from the brain of gilthead seabream (*Sparus aurata*) [24]. The cell line was cultivated in L-15 Leibowitz medium (Life Technologies, Waltham, MA, USA) supplemented with 10% fetal bovine serum (FBS, Life Technologies, Waltham, MA, USA), 2 mM glutamine (Life Technologies, Waltham, MA, USA), 100 μg mL^−1^ Streptomycin (Life Technologies, Waltham, MA, USA), 100 U mL^−1^ Penicillin (Life Technologies, Waltham, MA, USA) and 10 mM HEPES (Biowest, ID, Nampa, USA) in plastic tissue culture flasks (Nunc, ThermoFisher Scientific, Waltham, MA, USA) at 25 °C in an incubator with an atmosphere of 85% relative humidity. Cell cultures were subcultured by routine trypsinization methods. Cells were counted under a phase contrast microscope using the trypan blue exclusion test and viability results were always higher than 95%.

### 3.2. Nanoplastics Characterization

We used three types of commercial polystyrene nanoplastics (PS-NP; 50 nm): (PS-Plain) or functionalized with carboxyl (PS-COOH) or amine (PS-NH_2_) groups (Polysciences and Bangs Laboratories). Chemical composition of these NPs had been previously confirmed by Raman microspectroscopy analysis [65]. The hydrodynamic size and particle charge were measured, in triplicate samples, by Dynamic Light Scattering (DLS) (Zetasizer Nano ZS, Malvern instruments, UK) in both, ultrapure water (UW) and a complete culture medium following the protocol described in [10]. Particles were also examined by transmission electron microscopy (TEM; PHILIPS TECNAI 12, serial number D221, Philips, Netherlands), and the images were acquired with a digital camera (Megaview III, Olympus, Japan).

### 3.3. Exposure to Functionalized NPs

SaB-1 cells were seeded in well plates (Nunc), allowed to adhere overnight and then exposed for 24 h to NPs in order to evaluate toxicity, NP-cell interactions and the transcription of selected genes. To avoid NP agglomeration, stocks of NPs were vortexed for 30 s. A stock solution of NPs was prepared in UW (1 mg mL^−1^), further diluted in a culture medium and finally added to the culture wells to reach the desired concentrations. Controls contained the same diluents without NPs.

### 3.4. Cytotoxicity Assays

SaB-1 cells, seeded in 96-well plates at a density of 2.5 × 10^4^ per well, were exposed to 0.001 to 100 µg mL^−1^ of PS-NH_2_, PS-COOH or PS-Plain. Controls lacked NPs. The viability of cells was evaluated using the soluble tetrazolium salt (MTT) test [66] with a microplate reader (BMG Fluostar Omega, Labtech, Italy). Wells without cells were used as blanks. Exposure was performed in quadruplicate wells in at least three independent experiments.

The lethal concentration (LC) producing 10, 50 or 90% cell death was determined using the R software [67]. According to the MTT method, sublethal (1 μg mL^−1^; Sigma-Aldrich, St. louis, MO, USA) and LC_50_ (12 μg mL^−1^) dosages were used in the rest of assays.

### 3.5. Confocal Microscopy

SaB-1 cells were seeded on a coverslip and exposed to either 1 or 12 μg mL^−1^ of 50 nm red fluorescent PS-COOH (Micromer^®^-redF; excitation: 552 nm, emission: 580 nm; Micromod, Germany) for 24 h. Afterwards, cells were washed 3 times with 10 mM phosphate buffer (PBS, pH = 7.2–7.4), fixed using 4% paraformaldehyde and mounted in the coverslip using ProLong Gold with DAPI (ThermoFisher Scientific, Waltham, MA, USA). Samples were examined under a confocal laser scanning microscope (Leica SP8, Leica Microsystems, Mannheim, Germany) and images were obtained using a resonant scanner and a HC PLAPO CS2 63x/1.30 NA glycerol immersion lens by hybrid detector (Resolution: 1024 × 1024) and processed with Fiji software (National Institutes of Health, LOCI, University of Wisconsin, USA). 

### 3.6. Gene Expression Analysis

SaB-1 cells, seeded in 6-well microplates at 7.5 × 10^4^ cells well^−1^, were exposed for 24 h with a sublethal (1 µg mL^−1^) or 12 µg mL^−1^ (the LC_50_ for PS-NH_2_) dosages of NPs. Unexposed cultures served as controls. All treatments were performed and analysed in triplicates.

After exposure, the supernatant was aspirated and the total RNA isolated using the PureLink^®^ RNA Mini Kit (Life Technologies, Waltham, MA, USA), treated with DNAse I (Promega, Spain) to remove genomic DNA and the first strand of cDNA synthesized by reverse transcription using oligo dT and the SuperScript™ IV Reverse Transcriptase (Life Technologies, MA, USA). Real-time PCR was performed using 7500 Fast Real Time PCR System (Applied Biosystems, MA, USA) and PowerUp™ SYBR™ Green Master Mix (Applied Biosystems, MA, USA). Reaction mixtures were incubated at 95 °C for 10 min, followed by 40 cycles of 15 s at 95 °C, 1 min at 60 °C, and finally 15 s at 95 °C, 1 min at 60 °C and 15 s at 95 °C.

Gene expression was corrected by the geometric mean of the housekeeping elongation factor 1 alpha (*ef1a*) and ribosomal *18S* genes expression. Relative mRNA quantities of the target in each sample were normalized to the expression of the reference genes and to the unexposed or control cells following the 2^−∆∆CT^ method [68]. The primers are listed in Supplementary Table S1. Negative controls with no sample were always included in the reactions.

### 3.7. Combined Exposure to Functionalized NPs and Metals

The potential impact of NPs on the toxicity and transcriptomics of SaB-1 cells exposed to metals was evaluated as above. For this, two salts were tested: methylmercury (II) chloride [CH3HgCl] (MeHg); and arsenic trioxide (As_2_O_3_) (As) (Sigma–Aldrich, St. Louis, MO, USA). For the cytotoxicity evaluation, cells were exposed to metal concentrations ranging from 0.001 to 0.1 mM alone or in combination with a fixed concentration of 1 μg mL^−1^ NPs, which could be close to the worst environmental scenarios [47,69], and the viability assayed by the MTT as above. Controls lacked metals and NPs. For the transcriptomic study, SaB-1 cells were exposed to a sublethal (0.005 mM) and the LC_50_ dosage of the respective metal, alone or in combination with 1 µg mL^−1^ NPs, and the gene expression evaluated as above.

### 3.8. Data Analysis

Cytotoxic effects were presented using the fitted curves. Data were shown as mean ± SEM (*n* = 3). Statistical analysis was carried out using the SPSS v20 software. Two- and three-way ANOVA were performed with a Tukey’s post-hoc test for each response measured (*p* < 0.05). Levene’s test was considered in all analyses to check for homogeneity of the variance.

## 4. Conclusions

In summary, our results demonstrated that exposure of SaB-1 cells to functionalized NPs altered the transcription of genes related to metal protection, oxidative stress and apoptosis though only the PS-NH_2_ were toxic to the cells. Regarding exposure to metals, both As and MeHg were toxic to SaB-1 cells and induced metal detoxification, oxidative stress and apoptosis at the gene level. Finally, the presence of functionalized NPs increased the toxicity of the metals though changes at gene level were not evident. These results highlighted the potential hazard of functionalized NPs alone, or in combination with metals, and point to an added environmental risk.

## Figures and Tables

**Table 1:** Values of LC_10_, LC_50_, LC_90_ and R^2^ calculated after exposure of SaB-1 cells for 24 h to functionalized polystyrene nanoplastics (PS-NH_2_, PS-COOH and PS-Plain) or metals (As and MeHg) alone or metals in combination with 1 µg mL^−1^ of functionalized polystyrene nanoplastics (PS-NH_2_, PS-COOH and PS-Plain) (*n* = 3). LCs were calculated through interpolation of a nonlinear regression with a four-parameter dose-response curve. Student’s *t* test was applied to compare two fitted curves. *p*-value was established at 0.05. LC, lethal concentration; PS, polystyrene; NP, polystyrene nanoplastics; As, arsenic; MeHg, methylmercury; ND, not detected.

Treatment	LC_10_	LC_50_	LC_90_	R^2^	*p-*value *F (DFN.DFd)*
**NPs (µg mL^−1^) alone**					**Between NPs**
PS-NH_2_	1.67	12.59	95.92	0.97	**ND**
PS-COOH	***ND***	ND	ND	ND	
PS-Plain	***ND***	ND	ND	ND	
**Metals (mM) alone**					**Between metals**
As	0.011	0.041	0.140	0.90	***0.0115*** 7.017 (10.40)
MeHg	0.008	0.019	0.043	0.95	
**Metals + NPs combined**					**Metal vs. metal + NP**
As + PS-NH_2_	0.006	0.024	0.097	0.98	***0.0003*** 15.60 (10.40)
As + PS-COOH	0.005	0.023	0.097	0.98	***0.0018*** 11.16 (10.40)
As + PS-Plain	0.008	0.025	0.075	0.99	***0.006*** 8.425 (10.40)
MeHg + PS-NH_2_	0.005	0.013	0.038	0.99	***0.0001*** 18.72 (10.40)
MeHg + PS-COOH	0.005	0.012	0.036	0.99	***0.0001*** 19.64 (10.40)
MeHg + PS-Plain	0.008	0.02	0.05	0.99	0.620.239 (10.40)

**Table 2:** Summary of ANOVA results of the transcription of genes in SaB-1 cells upon 24 h exposure to 1 µg mL^−1^ or 12 µg mL^−1^ (LC_50_) of functionalized polystyrene nanoplastics (PS-NH_2_, PS-COOH and PS-Plain) alone, to sublethal or LC_50_ dosages of metals (As and MeHg) alone or to 1 µg mL^−1^ functionalized polystyrene nanoplastics in combination with sublethal or LC_50_ dosages of each metal (e.g., As + PS-COOH). Values of *p* < 0.05 are highlighted in italics. Asterisks (*) indicate the factors for which the interaction is evaluated. ANOVA, analysis of variance; LC, lethal concentration; NP, polystyrene nanoplastic; *mta*, Metallothionein A; *bcl2*, Bcl-2-associated X protein; *bax*, B-cell lymphoma 2; *nrf2*, Nuclear factor (erythroid-derived 2)-like 2; *cat*, Catalase; *gr*, Glutathione reductase; *gsta*, Glutathione S-transferase A.

Gene	Two-Way ANOVA						Three-Way ANOVA						
	NPs Alone			Metals Alone			NPs and Metals Combined						
	NPs	LC	NPs * LC	Metal	LC	Metal * LC	NPs	Metal	LC	NPs * Metal	NPs * LC	Metal * LC	NPs * Metal * LC
*mta*	*0.000*	*0.041*	*0.030*	*0.008*	*0.009*	*0.009*	0.902	*0.000*	*0.000*	0.848	0.729	*0.000*	0.895
*bcl2*	*0.002*	*0.039*	0.066	*0.005*	*0.018*	0.078	0.677	*0.000*	*0.002*	0.065	0.877	0.419	0.556
*bax*	0.311	0.496	0.943	*0.016*	0.561	0.177	0.592	0.104	0.351	0.259	0.886	0.971	0.780
*nrf2*	*0.030*	0.399	0.098	0.594	*0.000*	0.766	*0.020*	0.273	*0.000*	0.084	*0.000*	0.095	*0.039*
*cat*	0.677	*0.015*	0.233	0.081	0.054	0.006	0.495	0.098	*0.004*	0.642	0.371	0.107	0.433
*gr*	0.232	0.245	*0.021*	*0.005*	*0.001*	*0.032*	0.711	*0.000*	*0.010*	0.728	0.103	*0.033*	0.798
*gsta*	0.280	0.804	0.561	0.475	0.904	0.062	0.601	0.584	0.899	0.078	0.822	0.644	0.335

## Supplementary Materials

The following are available online at https://www.mdpi.com/article/10.3390/ijms22137141/s1, Table S1: Primers used for analysis of gene expression by real-time PCR in this study.
