# Supplementary material for: Functionalized Nanoplastics (NPs) Increase the Toxicity of Metals in Fish Cell Lines
Source: Int J Mol Sci. 2021 Jul 1;22(13):7141. doi: 10.3390/ijms22137141 (PMC8268098; doi:10.3390/ijms22137141)
Supplement: Supplementary file 1 [file ijms-22-07141-s001.zip › ijms-1262613-supplementary.pdf]

International Journal of Molecular Sciences

MDPI

Table S1. Primers used for analysis of gene expression by real-time PCR in this study.

Group	Protein Name	Gene		Sequence (5'→3')	Acc. number
House-keeping	Ribosomal protein S18	*rps18*	F	CGAAAGCATTTGCCAAGAAT	AM490061
			R	AGTTGGCACCGTTTATGGTC	
	Elongation factor 1α	*ef1a*	F	CTTCAACGCTCAGGTCATCAT	AF184170
			R	GCACAGCGAAACGACCAAGGGGA	
Metal protection	Metallothionein A	*mta*	F	ACAAACTGCTCCTGCACCTC	X97276
			R	CAGCTAGTGTCGCACGTCTT	
Apoptosis	Bcl-2-associated X protein	*bax*	F	CAACAAGATGGCATCACACC	AM963390
			R	TGAACCCGCTCGTATATGAAA	
	B-cell lymphoma 2	*bcl2*	F	TCAGGAGTGATGTCGAGCTG	FM145663
			R	CAGCCAGGTGCTGACATAGA	
Oxidative stress	Nuclear factor (erythroid-derived 2)-like 2	*nrf2*	F	GTTCAGTCGGTGCTTTGACA	FP335773
			R	CTCTGATGTGCGTCTCTCCA	
	Catalase	*cat*	F	TTCCCGTCCTTCATTCACTC	FG264808
			R	CTCCAGAAGTCCCACACCAT	
	Glutathione reductase	*gr*	F	CAAAGCGCAGTGTGATTGTGG	AJ937873
			R	CCACTCCGGAGTTTTGCATTTC	
	Glutathione S-transferase A	*gsta*	F	AAGACTTCCGGTGGTTTCCT	XM_030413028
			R	GTAACGCTCCTCGCATAACC	
